# Correction for Christensen et al., “Identification of Novel Protein Lysine Acetyltransferases in Escherichia coli”

**DOI:** 10.1128/mBio.00592-19

**Published:** 2019-04-09

**Authors:** David G. Christensen, Jesse G. Meyer, Jackson T. Baumgartner, Alexandria K. D’Souza, William C. Nelson, Samuel H. Payne, Misty L. Kuhn, Birgit Schilling, Alan J. Wolfe

**Affiliations:** a Department of Microbiology and Immunology, Stritch School of Medicine, Health Sciences Division, Loyola University Chicago, Maywood, Illinois, USA; b Buck Institute for Research on Aging, Novato, California, USA; c Department of Chemistry and Biochemistry, San Francisco State University, San Francisco, California, USA; d Biological Sciences Division, Pacific Northwest National Laboratory, Richland, Washington, USA

## AUTHOR CORRECTION

Volume 9, issue 5, e01905-18, 2018, https://doi.org/10.1128/mBio.01905-18. We correct the following errors in our publication. In two instances, we cited the wrong reference.

For the sentence below, from PDF page 11:

“For instance, E103 coordinates a water molecule to act as a general base in RimI from Salmonella enterica serovar Typhimurium LT2 (21)…”

Instead of reference 21, we should have cited the following reference.

Vetting MW, Bareich DC, Yu M, Blanchard JS. 2009. Crystal structure of RimI from *Salmonella typhimurium LT2*, the GNAT responsible for N^α^-acetylation of ribosomal protein S18. Protein Sci 17:1781–1790. https://doi.org/10.1110/ps.035899.108.

For the sentence below, from PDF page 16:

“This is an interesting observation, as RimI from both E. coli and *Salmonella* Typhimurium is known to function as an N-terminal alanine acetyltransferase that has but one known target, the ribosomal protein S18 (21, 48, 49).”

Reference 21 should be replaced by the Vetting et al. reference listed above.

