# Supplementary figures and images for: Identification of Novel Protein Lysine Acetyltransferases in Escherichia coli
Source: mBio. 2018 Oct 23;9(5):e01905-18. doi: 10.1128/mBio.01905-18 (PMC6199490; doi:10.1128/mBio.01905-18)

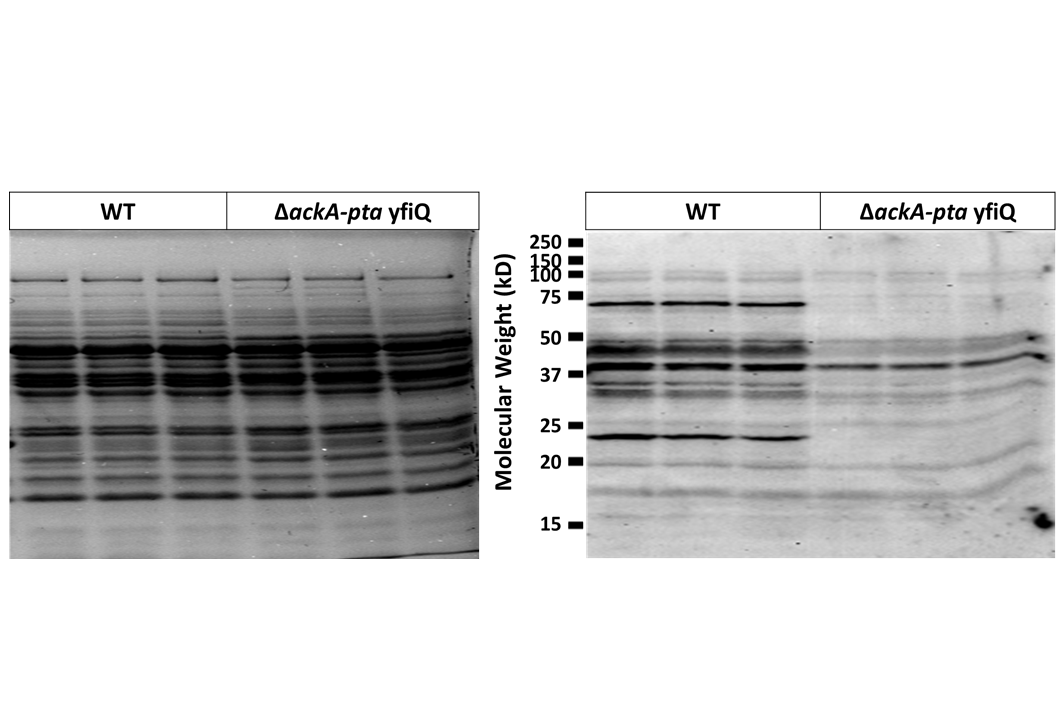

Supplement: FIG S1 [file mbo005184126sf1.tif]

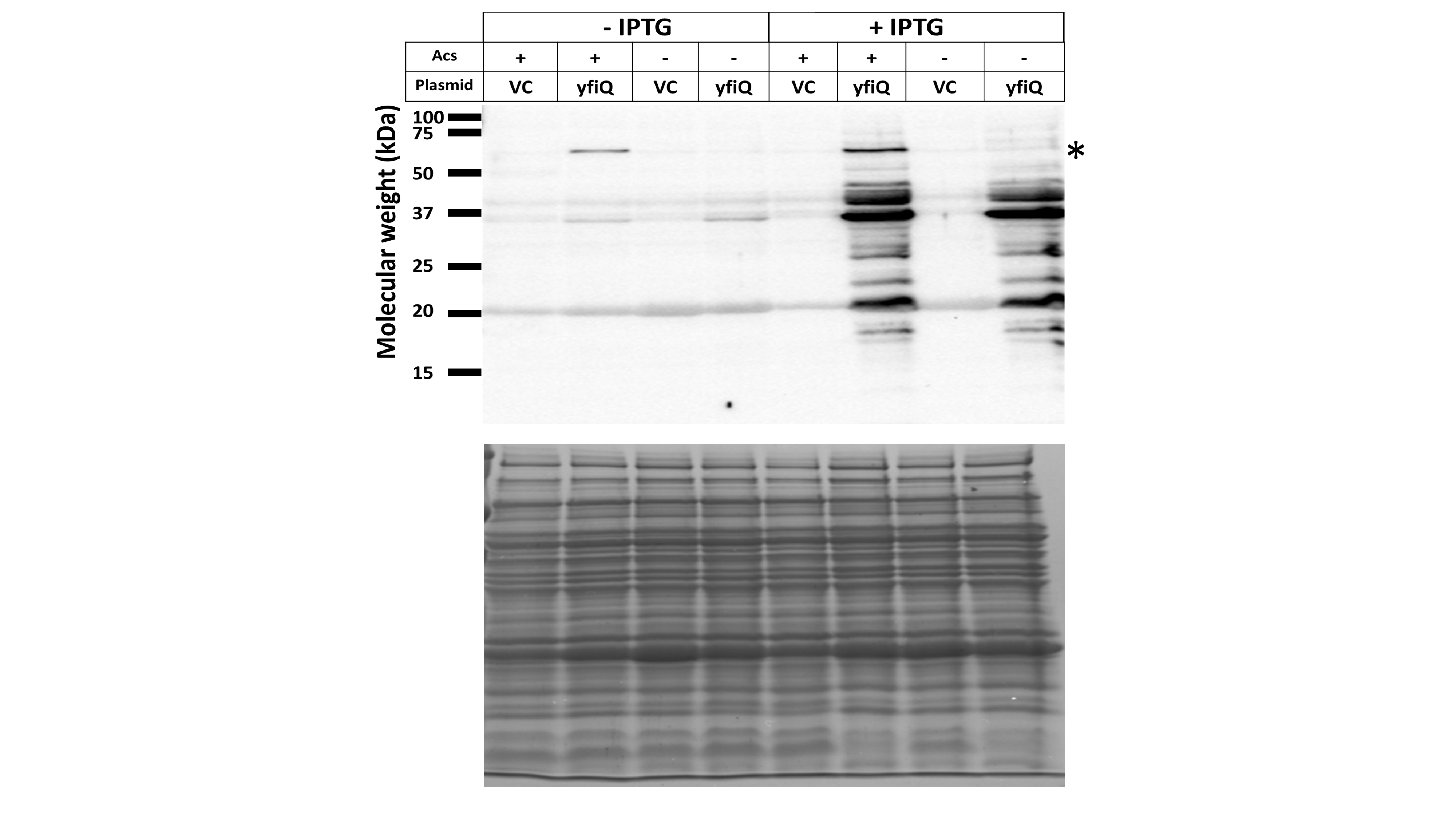

Supplement: FIG S2 [file mbo005184126sf2.tif]

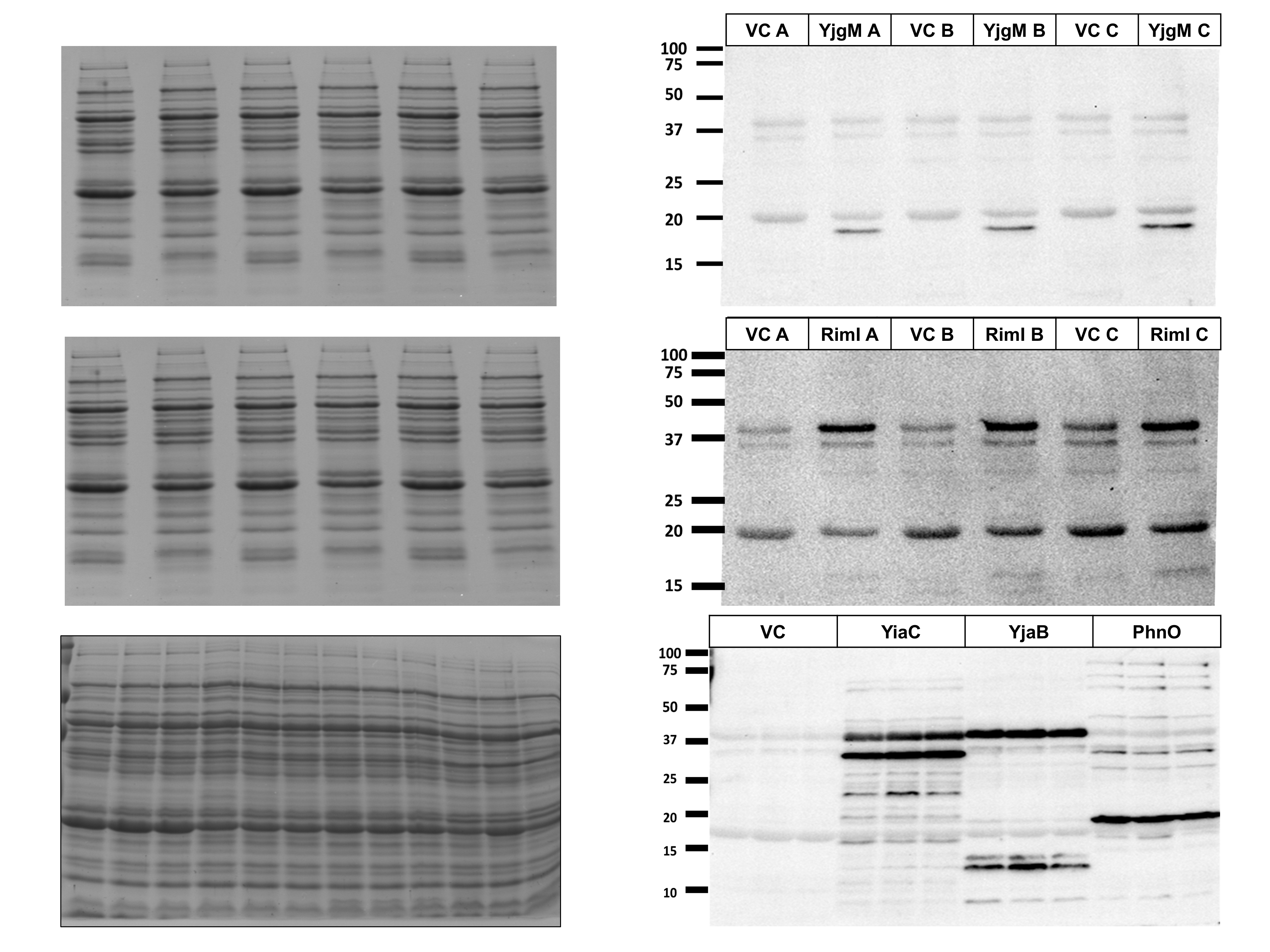

Supplement: FIG S3 [file mbo005184126sf3.tif]

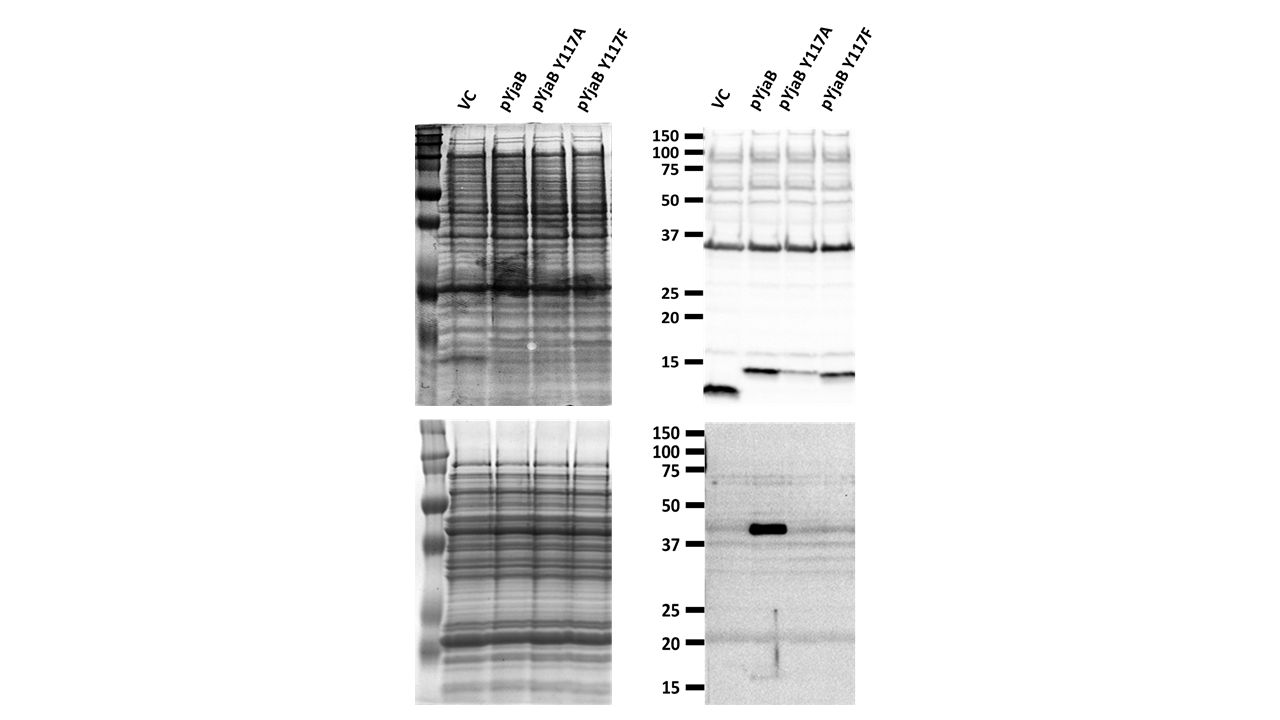

Supplement: FIG S4 [file mbo005184126sf4.tif]

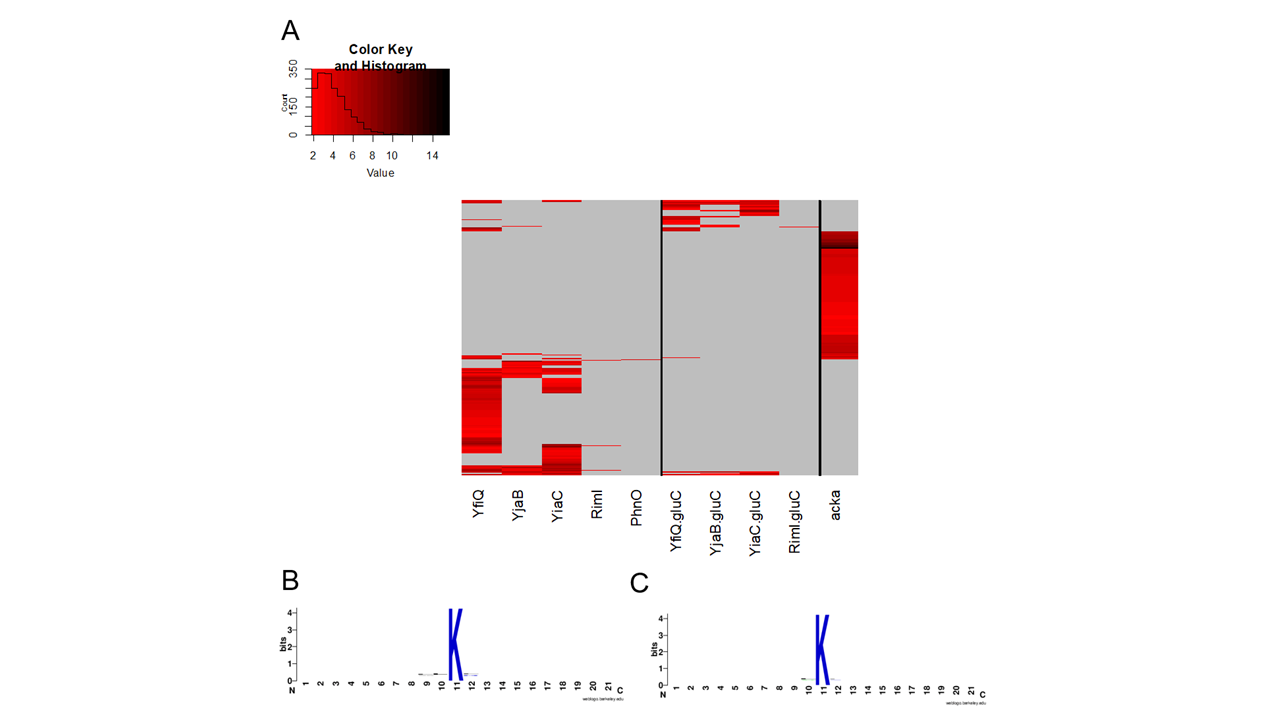

Supplement: FIG S5 [file mbo005184126sf5.tif]

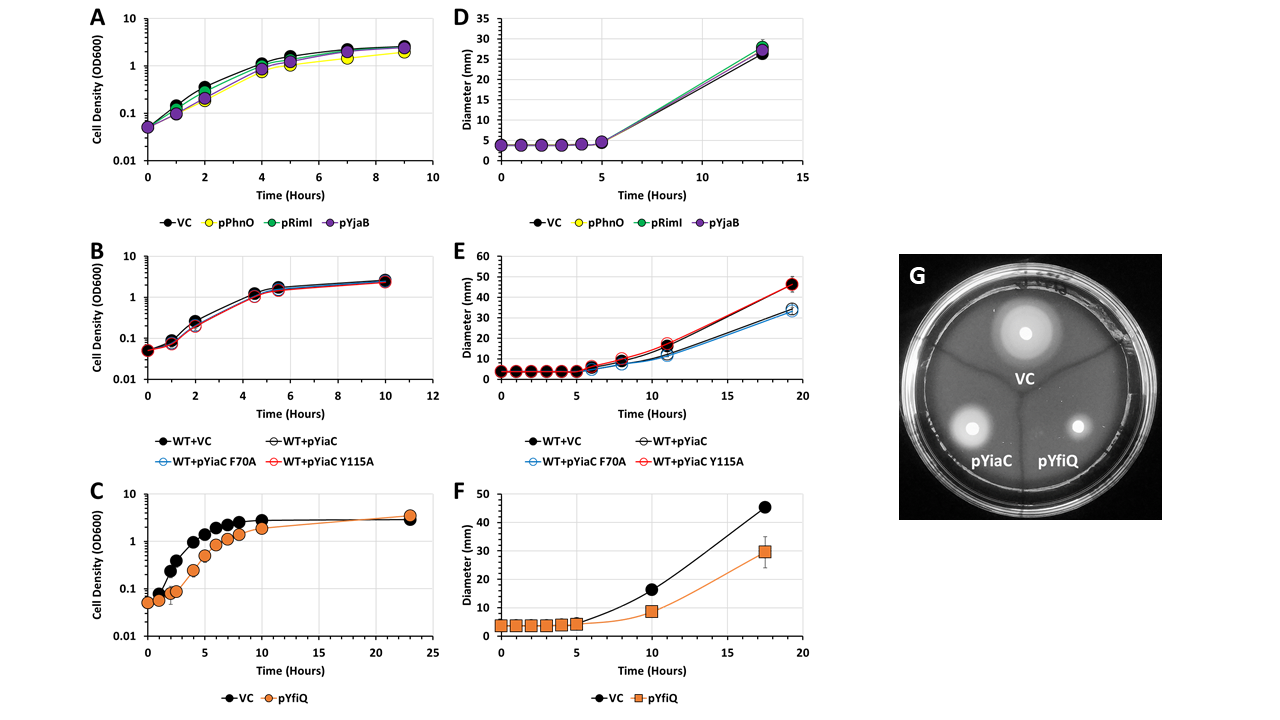

Supplement: FIG S6 [file mbo005184126sf6.tif]
